# Comparison of Post-Transplantation Lymphoproliferative Disorder Risk and Prognostic Factors between Kidney and Liver Transplant Recipients

**DOI:** 10.3390/cancers14081953

**Published:** 2022-04-13

**Authors:** Krzysztof Mucha, Rafał Staros, Bartosz Foroncewicz, Bogna Ziarkiewicz-Wróblewska, Maciej Kosieradzki, Sławomir Nazarewski, Beata Naumnik, Joanna Raszeja-Wyszomirska, Krzysztof Zieniewicz, Leszek Pączek

**Affiliations:** 1 Department of Immunology, Transplantation and Internal Medicine, Medical University of Warsaw, 02-006 Warsaw, Poland; rafal.j.staros@gmail.com (R.S.); bartosz.foroncewicz@wum.edu.pl (B.F.); leszek.paczek@wum.edu.pl (L.P.); 2 Institute of Biochemistry and Biophysics, Polish Academy of Sciences, 02-106 Warsaw, Poland; 3 University Clinical Center, Medical University of Warsaw, 02-091 Warsaw, Poland; wroblewskabogna@o2.pl; 4 Department of General and Transplant Surgery, Medical University of Warsaw, 02-006 Warsaw, Poland; mkosieradzki@wum.edu.pl; 5 Department of General, Vascular and Transplant Surgery, Medical University of Warsaw, 02-097 Warsaw, Poland; slawomir.nazarewski@wum.edu.pl; 6 1st Department of Nephrology and Transplantation with Dialysis Unit, Medical University of Białystok, 15-540 Białystok, Poland; bnaumnik@poczta.onet.pl; 7 Liver and Internal Medicine Unit, Medical University of Warsaw, 02-097 Warsaw, Poland; jorasz@gmail.com; 8 Department of General, Transplant and Liver Surgery, Medical University of Warsaw, 02-097 Warsaw, Poland; krzysztof.zieniewicz@wum.edu.pl

**Keywords:** kidney transplantation, liver transplantation, post-transplant lymphoproliferative disorder, solid organ transplantation

## Abstract

**Simple Summary:**

Organ graft type is an independent risk factor for the development of PTLD. Due to small cohort sizes caused by the rarity of the disease, most studies and trials are performed on populations of recipients of various organ graft types. Our study is a first direct comparison of the effect of different risk and prognostic factors between kidney and liver transplant recipients (KTRs and LTRs, respectively). We have demonstrated that the analysis of risk factors based on the general solid organ transplant recipient population is inconsistent with the results obtained through the analysis of single organ graft type cohorts. The risk of PTLD is lower in KTRs who are female, <45 years old at transplantation, or treated with cyclosporin. Based upon our findings, we are confident that the type of organ graft is an overriding factor in PTLDs’ development and course.

**Abstract:**

Post-transplantation lymphoproliferative disorder (PTLD) is a life-threatening complication of solid organ transplantation (SOT). Its development risk varies among organ graft recipients. In this study, retrospective data were analyzed to compare PTLD’s risk and prognostic factors between adult kidney and liver transplant recipients (KTRs and LTRs, respectively). Over 15 years, 2598 KTRs and 1378 LTRs were under observation at our center. Sixteen KTRs (0.62%) and twenty-three LTRs (1.67%) were diagnosed with PTLD. PTLD developed earlier in LTRs (*p* < 0.001), SOT patients > 45 years old (*p* = 0.002), and patients receiving tacrolimus (*p* < 0.001) or not receiving cyclosporin (*p* = 0.03) at diagnosis. Tacrolimus use, male sex, and age > 45 years old significantly affected the time of PTLD onset in KTRs (hazard ratio (HR) = 18.6, 7.9 and 5.2, respectively). Survival was longer in LTRs < 45 years old (*p* < 0.009). LTRs were more likely than KTRs to achieve complete remission (*p* = 0.039). Factors affecting PTLD development and outcome differ between KTRs and LTRs; thus, these populations should be separately evaluated in future studies.

## 1. Introduction

Post-transplantation lymphoproliferative disorder (PTLD) was first described as a life-threatening complication of allotransplantation in 1969 by Penn et al. [1] in a series of five kidney graft recipients diagnosed with lymphoma. Currently, it is recognized as a heterogeneous group of lymphoid neoplasms occurring in hematopoietic stem cell and solid organ transplant (SOT) recipients. These groups of patients are at increased risk of carcinogenesis, with standardized incidence ratios nearing 10 for non-Hodgkin lymphoma (NHL) and 4 for classic HL [2]. According to the 2016 revision of the World Health Organization (WHO) classification of lymphoid neoplasms [3], the following histopathological PTLD subtypes are recognized: infectious mononucleosis, plasmacytic hyperplasia, florid follicular hyperplasia, polymorphic, monomorphic (B- and T/natural killer (NK)-cell subtypes with the exclusion of indolent lymphomas), and HL. Previous research has established a clear role of Epstein-Barr virus (EBV) in the development of early-onset PTLD (<12 months after transplantation), especially in pediatric graft recipients. It has been reported that primary EBV infection combined with the effects of T-cell depleting induction and maintenance immunosuppression (IS) can drive malignant B-cell transformation [4]. However, the pathogenesis of PTLD is multifactorial and not entirely understood, as in up to 50% of all cases, the disease is EBV-negative [5], up to 10% of neoplasms stem from T/NK cells [6,7], and another peak of onset is observed at 10–15 years post-transplantation [8,9]. To date, several other risk factors have been proposed or identified based on varying degrees of evidence [10], including the cumulative dose of immunosuppressants (induction, maintenance, and acute rejection treatment), use of certain immunosuppressive agents, primary diseases [11], and viral infections such as cytomegalovirus (CMV), hepatitis B (HBV), and C (HCV) [12,13,14,15].

Assembling a large cohort of single type organ graft patients with PTLD is difficult; therefore, most researchers have investigated PTLD in combined populations of different organ graft recipients, and found that the relative risk (RR) of disease development significantly varies according to the type of transplanted organ [16,17,18,19,20,21]. Multiorgan and intestinal graft recipients have the highest RR (RR = 239.5) in contrast to lung transplant recipients (RR = 58.6), pancreas transplant recipients (RR = 34.9), liver transplant recipients (LTRs) (RR = 29.9), heart transplant recipients (RR = 27.6), and kidney transplant recipients (KTRs) (RR = 12.6) [22]. 

This study identified the factors contributing to the development, course, response to treatment, and outcomes of PTLD and compared their impact on KTRs and LTRs.

## 2. Materials and Methods

### 2.1. Study Participants 

This retrospective cohort study included 39 patients diagnosed with PTLD out of 2598 KTRs and 1378 LTRs followed up at our center between 2002 and 2017. The KTR group was defined as KTRs and non-simultaneous KTRs and LTRs who received a kidney graft first. The LTR group was defined analogically. The SOT group consisted of 39 patients (KTRs and LTRs). Both KTRs and LTRs included one non-simultaneous KTR and LTR each. No pediatric patients were included in the study; however, three KTRs and one LTR underwent transplantation at the age of 6–14 years and developed PTLD in adulthood. Identified cases were classified into PTLD subtypes according to the 2016 revision of the WHO classification of lymphoid neoplasms. In PTLD cases confirmed posthumously, the date of first cytological or imaging results suggestive of PTLD was considered the date of PTLD diagnosis. EBV positivity was determined to be either active EBV replication or the presence of latent membrane protein 1 in histopathological samples. The analyzed data were collected from the patients’ medical records. This study was approved by The Bioethics Committee of the Medical University of Warsaw, approval No. AKBE/90/2022.

### 2.2. Statistical Analyses

The statistical differences in clinical characteristics were assessed using the Mann–Whitney U test. The Welch’s *t*-test was performed to compare the mean parameters, and the log-rank test was used to compare Kaplan–Meier estimator curves between groups. Univariate and multivariate analyses of the effects of variables on the PTLD time of onset were calculated using the Cox proportional hazards model and Wald test. Data analyses were performed in R version 3.2.3. *p* < 0.05 was considered statistically significant.

## 3. Results

### 3.1. Clinical Characteristics

The clinical characteristics are summarized in Table 1. The prevalence of PTLD was 0.62% (16 patients) and 1.67% (23 patients) in KTRs and LTRs, respectively. The incidence rate of PTLD was 0.8 per 1000 patient-years for KTRs and 2.1 per 1000 patient-years for LTRs. There was no significant difference in sex distribution among the PTLD patients. The median age at first transplantation was 28 years for KTRs (range 6–70 years) and 48 for LTRs (range 12–60). 

Antibody induction was used in a total of 16 SOT recipients (41.03%). Anti-cluster of differentiation 25 (CD25) antibodies were administered to 12 of 23 LTRs (52.17%) and 3 of 16 KTRs (18.75%). Anti-thymocyte globulin was used in only one KTR (6.25%). A history of acute rejection was recorded in one-third of patients in both groups. Four LTRs received tacrolimus (TAC) monotherapy as maintenance IS. The remaining patients were either on two- or three-drug regimens composed of glucocorticosteroids, calcineurin inhibitor (cyclosporin (CsA) or TAC), and an anti-proliferative agent (azathioprine (AZA) or mycophenolate mofetil (MMF)). Compared to LTRs, a significantly larger number of KTRs received regimens containing CsA and AZA, whereas TAC-based IS was used in a significantly higher proportion of LTRs than KTRs (Supplement Table S1). No significant differences in viral replication and serological status for CMV, HBV, and HCV were detected. Only a difference in EBV positivity approached statistical significance (*p* = 0.053); however, in 31.25% of KTRs and 21.74% of LTRs, the data regarding EBV were unavailable. PTLD was mostly diagnosed at >12 months after transplantation (late onset), and its type was not significantly different between LTRs and KTRs (Table 2). Data regarding the location and dissemination of PTLD are presented in Supplement Table S2.

### 3.2. Factors Associated with PTLD Development

**Figure 1:**
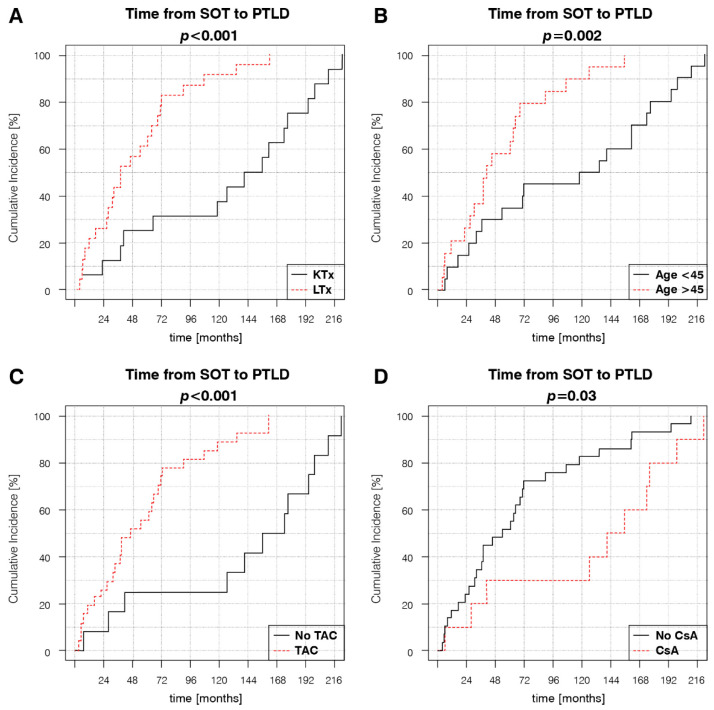
Factors influencing the dynamic of PTLD development in the SOT group: (**A**) organ type, (**B**) age at transplantation, (**C**) use of TAC in the maintenance IS regimen at the time of PTLD diagnosis, and (**D**) use of cyclosporin in maintenance IS at the time of PTLD diagnosis. *p*-values represent the log-rank analysis of the Kaplan–Meier estimator curves.

**Figure 2:**
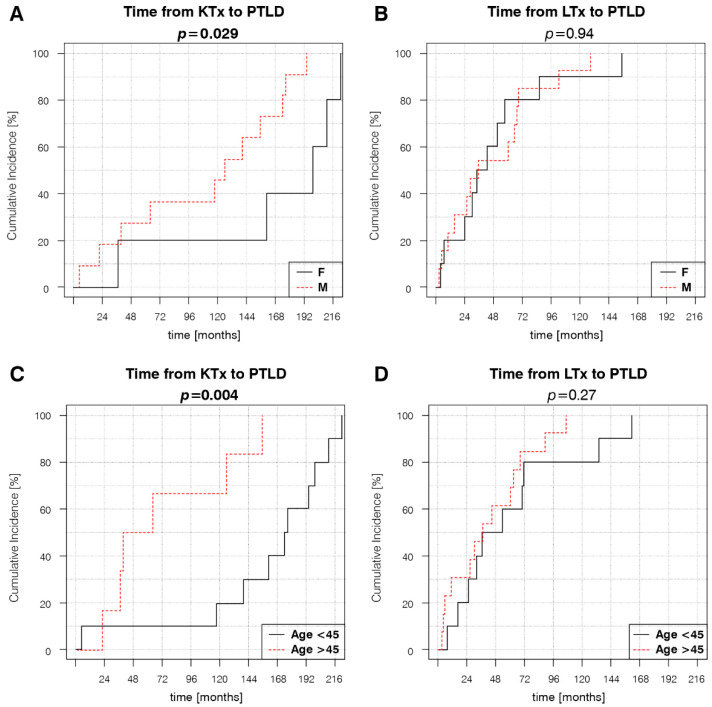

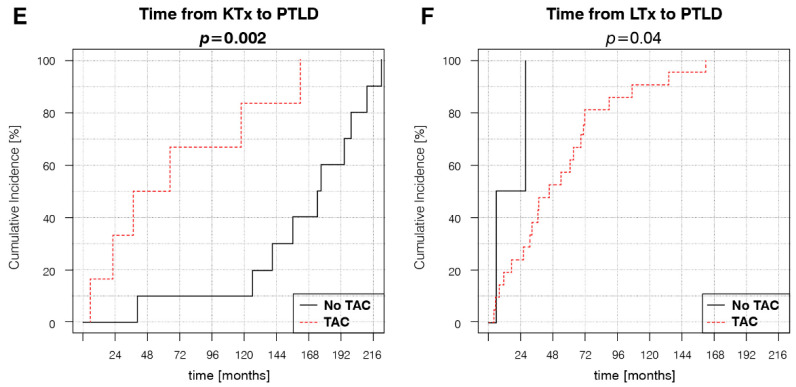
Transplanted organ-dependent effect of different variables on PTLD development: (**A**) KTR sex, (**B**) LTR sex, (**C**) KTR age at transplantation, (**D**) LTR age at transplantation, (**E**) Presence of TAC in KTR IS regimens at PTLD diagnosis, (**F**) Presence of TAC in LTR IS regimens at PTLD diagnosis. *p*-values represent the log-rank analysis of the Kaplan–Meier estimator curves.

Kaplan–Meier estimator curves were plotted and compared using the log-rank test to determine the impact of different variables on the time of PTLD onset. The analyses revealed significantly different dynamics of disease development between KTRs and LTRs, with a mean time to PTLD diagnosis of 118.6 months (9.9 years) and 55.8 months (4.7 years), respectively (Figure 1A). Earlier PTLD development in the SOT population was associated with age at transplantation > 45 years, TAC-based, and non-CsA IS regimens (Figure 1B–D). Subsequent Kaplan–Meier estimator curve analyses for separate KTR and LTR groups showed that the significance of the aforementioned risk factors changed in relation to the type of transplanted organ. Age at transplantation > 45 years retained significance in KTRs, but not in LTRs (Figure 2C,D). TAC-based IS regimens retained significance in both KTRs and LTRs (Figure 2E,F). Female sex, a factor that was not significant in the SOT group, was significantly associated with later PTLD onset in the KTRs (Figure 2A,B).

The results obtained through log-rank analyses of the Kaplan–Meier estimator curves were verified using univariate and multivariate Cox proportional hazards models (Table 3). In the SOT group, univariate analyses revealed a significantly increased hazard ratio (HR) of earlier PTLD development in patients older than 45 years at transplantation (HR = 1.03, 95% confidence interval (CI) = 1.01–1.05; *p* = 0.006), patients with TAC-based IS (HR = 5.09, 95% CI = 2.00–12.95; *p* < 0.001), and a decreased HR in patients treated with CsA-based IS regimens (HR = 0.44, 95% CI = 0.20–0.95; *p* = 0.036). In univariate analyses, significant increases in HR were detected in KTRs > 45 years old at transplantation (HR = 1.04, 95% CI = 1.00–1.07; *p* = 0.044), male KTRs (HR = 4.86, 95% CI = 1.03–22.81; *p* = 0.045), and KTRs receiving TAC (HR = 6.25, 95% CI = 1.68–23.21; *p* = 0.006).

In multivariate analyses, only age at transplantation > 45 years remained a significant risk factor for all SOT patients (HR = 3.16, 95% CI = 1.4–7.1; *p* = 0.005). For KTRs, all three factors retained significance: age > 45 years at transplantation (HR = 5.21, 95% CI = 1.04–26.25; *p* = 0.045), male sex (HR = 7.87, 95% CI = 1.25–49.26; *p* = 0.027), and TAC-based IS (HR = 18.57, 95% CI = 2.79–123.87; *p* = 0.003).

### 3.3. Treatment and Outcomes

**Figure 3:**
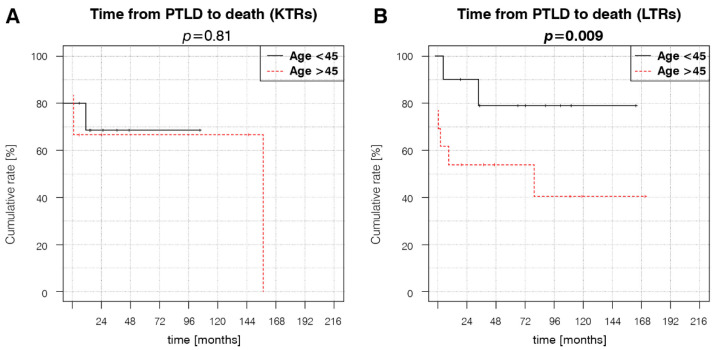
Factors influencing patient survival. (**A**) KTR age at transplantation, (**B**) LTR age at transplantation. *p*-values represent the log-rank analysis of the Kaplan–Meier estimator curves.

Both groups received similar PTLD treatment (Table 4). LTRs were more likely to achieve complete remission (CR) than KTRs, whereas other outcomes did not significantly differ between the groups (Table 5). Kaplan–Meier estimator curve analyses revealed that patient survival was superior in LTRs < 45 years old at transplantation (Figure 3B). In KTRs, age did not significantly affect survival (Figure 3A).

## 4. Discussion

We identified the effect of specific IS agents on PTLD development in KTR and LTR populations. Our findings that CsA use or TAC avoidance in KTRs in female patients younger than 45 years are associated with later PTLD onset support the idea of tailored maintenance IS regimens as a risk-reduction strategy. These findings contradict the current consensus that the intensity and cumulative dose of IS, rather than particular maintenance immunosuppressive agents, affect PTLD development and course. Prospective validation of these findings is warranted.

We demonstrated that the significance of risk factors is dependent on the type of transplanted organ. Thus, extrapolation of the impact of PTLD risk factors in combined SOT recipient cohorts onto graft-specific populations may produce inaccurate results. For example, none of the three significant variables affecting the time of PTLD onset in our total SOT cohort retained significance in the LTR subgroup, and only two of three remained significant in the KTR group. Transplanted organ-related differences in the time of PTLD onset were recently reported by Lau et al. [23]. The authors found that the median time from transplantation to diagnosis was 0.49 years for LTRs and 4.0 years for KTRs, whereas, in our study, the mean time to PTLD diagnosis was 4.7 and 9.9 years for LTRs and KTRs, respectively. Such a difference may be attributed to the high proportion of pediatric patients in the cohort of Lau et al., as they require more IS and have a higher incidence of primary EBV infection than adults. Furthermore, a higher PTLD incidence among LTRs compared to KTRs in our cohort is in accordance with previous reports [24].

KTRs > 50 years of age at transplantation have an increased risk of late-onset PTLD (HR = 1.28) [25]. Our results show a marked risk increase (HR = 5.21) at a younger cut-off age of 45 years at kidney transplantation. Additionally, age > 55 years was previously reported to negatively impact the survival of KTRs with PTLD [26], which is not supported by our data in the KTR group. We consider the improved survival of LTRs < 45 years at liver transplantation a novel finding, as most studies analyzing the prognostic factors for LTRs are based on pediatric or mainly pediatric cohorts and describe an inverted age–PTLD association, where adult recipients demonstrate improved survival. In a paper by Fararjeh et al. [27], age was not a significant factor in the survival of adult LTRs.

To date, data on the role of patients’ sex in PTLD development in SOT recipients are limited. Moreover, sex analyses are mostly associated with patient survival [28]. The impact of different immunosuppressive agents on the PTLD time of onset and patients’ survival has been a subject of debate. Large registry studies have shown contradictory effects of TAC use with PTLD development in KTRs: Bustami et al. reported an increased risk of PTLD in first-time KTRs without antibody induction [29], whereas Francis et al. described a decreased risk of PTLD [30]. Additionally, a combination of MMF and CsA use is associated with a lower PTLD risk in KTRs [31]. Despite the statistical significance of TAC use in later PTLD onset among LTRs, it must be underlined that 21 out of 23 patients in this group received TAC and only 2 received CsA. However, the *p*-value of this analysis was *p* = 0.04. This observation should be validated on larger cohorts of LTRs taking TAC or CsA. Additionally, the significantly higher number of patients treated with TAC in the LTR group, who tend to develop PTLD earlier than KTRs in our cohort, might have affected the Kaplan–Meier estimator curve analysis of the effect of TAC on the time to PTLD diagnosis in the SOT group. We consider this as a possible limitation of this analysis.

The outcomes of PTLD treatment, particularly the 54% patient survival and the lack of a clear, statistically significant advantage of any treatment modality at this sample size, may be a result of the inclusion of patients treated before the rituximab era and of the high percentage of monomorphic T-cell PTLD cases. Initial reduction in IS treatment (RIS) was performed in 36 of 39 patients in our study and is concurrent with reports of withdrawal or reduction in AZA, MMF, or CNI or even temporary cessation of maintenance IS during chemotherapy [32]. However, the clinical outcome of RIS is highly variable and depends on the type and lineage of PTLD [33]. Contemporary treatment strategies yield higher efficacy, as confirmed by the results of the PTLD-1 prospective, international, multicenter phase II clinical trial. In that study, 88% of B-cell derived PTLD patients demonstrated a complete or partial response to rituximab induction, followed by rituximab monotherapy or an immunochemotherapy consolidation regimen consisting of rituximab, cyclophosphamide, doxorubicin, vincristine, and prednisone (R-CHOP) [34]. The higher frequency of CR in the LTR than in KTR group in our study may be due to a relatively high number of non-diffuse large B-cell lymphoma monomorphic B-cell PTLD (e.g., lymphomatoid granulomatosis, plasmacytic myeloma) and monomorphic T-cell PTLD among KTRs. The observed higher rate of T-cell PTLD in KTRs is in accordance with previous studies—in their meta-analysis of 9 local and 147 reported cases of T-cell PTLD, Herreman and Dierickx found that this subtype of PTLD is most frequent in KTRs, who constituted 63% of all cases [35]. Although statistically insignificant, the higher number of PH PTLD patients in LTRs may reflect the shorter time to PTLD development in this group and lower intensity of IS treatment.

Recently, evidence emerged that the epidemiology of PTLD is evolving, and with current antiviral prophylaxis and stricter donor matching for EBV-seronegative recipients, there is an observable trend towards an increase in late-onset, EBV-negative PTLD [36,37,38,39]. Conversely, the field of oncological and immunological research in the immunocompromised population is expanding, as novel clinicopathological entities such as EBV-positive mucocutaneous ulcer [40] are still being discovered, and new modes of therapy are being proposed. Regarding PTLD patients, the most promising results are from studies investigating adoptive immunotherapy such as EBV-specific cytotoxic T cells [41] and chimeric antigen receptor T cells [42,43]. Both treatments can provide a sustained anti-PTLD response by T-cell engraftment, reducing the risk of disease relapse and serving as an end of the line intervention for rituximab or R-CHOP non-responders [44,45]. Recently, various NK-cell therapies have been suggested as possible PTLD treatment based on studies conducted on immunocompetent non-Hodgkin lymphoma patients [46]. In our study, no significant differences in the viral DNA and serological status for EBV were detected.

To the best of our understanding, this study had two main limitations. First, the sample size was relatively small, leading to wide CIs in the multivariate analyses. Second, the data regarding EBV status were missing in one-quarter of the patients, resulting in a nearly significant discrepancy in KTR and LTR group composition. However, the major advantages of our study were the 15-year follow-up period of a large cohort of 3976 SOT recipients and homogenous post-transplant medical care.

## 5. Conclusions

In summary, CsA use in female KTRs < 45 years at transplantation postpones PTLD development. Despite the cohort size, multivariate analyses supported this claim, as statistically significant changes in HR were found for these variables. The data suggest that graft type might be an overriding risk determinant, as the dynamics of PTLD onset and the impact of multiple other risk and prognostic factors significantly differ in relation to the type of transplanted organ. Further analyses are necessary to identify subsets of patients who might benefit from specific maintenance treatment modalities. Such an approach seems invaluable to achieve tailored diagnostic tools and treatment modalities for SOT recipients.

## Figures and Tables

**Table 1:** Clinical characteristics.

	LTRs	KTRs	
Patient Characteristics	*n* (%)		*p*-Value
Number of patients	23 (100)	16 (100)	
PTLD incidence rate (per 1000 patient-years) Mean age at Tx (years) [SD]	2.1	0.8	
	43.7 (12.0)	36.3 (20.7)	0.21
Males Females	13 (56.5)	11 (68.8)	0.456
	10 (43.5)	5 (31.3)	0.456
KT+LTRs Retransplantation	1 (4.4)	1 (6.3)	1.0
	1 (4.4)	1 (6.3)	1.0
IS Treatment			
Induction			
Anti-CD25	12 (52.2)	3 (18.8)	0.076
ATG	0 (0)	1 (6.3)	0.853
Acute rejection			
Total	8 (34.8)	5 (31.3)	0.329
	1 (4.4) no data	2 (12.5) no data	
GCS treated	8 (34.8)	5 (31.3)	0.984
ATG treated	1 (4.4)	0 (0)	0.456
Maintenance			
Monotherapy	4 (17.4)	0 (0)	0.052
Double drug therapy	12 (52.2)	6 (37.5)	0.381
Triple drug therapy	7 (30.4)	10 (62.5)	0.052
GCS	14 (60.9)	13 (81.3)	0.428
CsA	2 (8.7)	8 (50)	0.004 *
TAC	21 (91.3)	6 (37.5)	<0.001 *
AZA	1 (4.4)	5 (31.3)	0.025 *
MMF	11 (47.8)	10 (62.5)	0.255
Viral Replication	LTRs	KTRs	*p*-Value
EBV DNA	10 (43.2)	10 (62.5)	0.053
	5 (21.7) no data	5 (31.3) no data	
CMV DNA	0 (0)	2 (12.5)	0.093
HBV DNA	2 (8.7)	1 (6.3)	0.805
HCV RNA	5 (21.7)	1 (6.3)	0.201

ATG—anti-thymocyte globulin, AZA—azathioprine, CMV—cytomegalovirus, CsA—cyclosporin, EBV—Epstein-Barr virus, GCs—glucocorticosteroids, HBV—hepatitis B virus, HCV—hepatitis C virus, KT+LTRs—non-simultaneous kidney and liver transplant recipients, KTRs—kidney transplant recipients, LTRs—liver transplant recipients, MMF—mycophenolate mofetil, TAC—tacrolimus (Mann–Whitney U-test for comparison between KTRs and LTRs), * *p* < 0.05.

**Table 2:** PTLD characteristics.

	LTRs	KTRs	
PTLD Characteristics	*n* (%)		*p*-Value
Focal disease	11 (47.8)	8 (50)	0.908
Disseminated disease Early onset	12 (52.2)	8 (50)	0.908
	5 (21.7)	1 (6.3)	0.16
Late onset Post-mortem diagnosis	18 (78.3)	15 (93.8)	0.16
	3 (13)	0 (0)	0.083
WHO 2016 Type			
Florid follicular hyperplasia	1 (4.4)	0 (0)	0.434
Infectious mononucleosis	0 (0)	1 (6.3)	0.251
Plasmacytic hyperplasia	6 (26.1)	1 (6.3)	0.122
Polymorphic	4 (17.4)	1 (6.3)	0.324
Monomorphic B-cell	11 (47.8)	9 (56.3)	0.182
-DLBCL	9 (39.1)	3 (18.8)	0.168
-HGBL	1 (4.4)	1 (6.3)	0.805
-Lymphomatoid granulomatosis	0 (0)	2 (12.5)	0.164
-Plasmacytic myeloma	0 (0)	1 (6.3)	0.333
-MCL	0 (0)	1 (6.3)	0.333
-Other	1 (4.4)	1 (6.3)	0.805
Monomorphic T-cell	1 (4.4)	3 (18.8)	0.167
Classic Hodgkin lymphoma	0 (0)	1 (6.3)	0.853

Early onset is <12 months after transplantation, late onset is >12 months after transplantation, DLBCL—diffuse large B-cell lymphoma, HGBL—high-grade B-cell lymphoma, KTRs—kidney transplant recipients, LTRs—liver transplant recipients, MCL—mantle cell lymphoma. *p*-values represent the Mann–Whitney U-test comparison between KTRs and LTRs.

**Table 3:** Univariate and multivariate Cox proportional hazards models of the effect of variables on the PTLD time of onset.

SOT—Univariate				KTR—Univariate				LTR—Univariate			
Variable	HR	95% CI	*p*-Value	Variable	HR	95% CI	*p*-Value	Variable	HR	95% CI	*p*-Value
Male sex	1.49	(0.74–3.02)	0.269	Male sex	4.86	(1.03–22.81)	0.045 *	Male sex	1.03	(0.44–2.44)	0.939
Age at Tx > 45 years	1.03	(1.01–1.05)	0.006 *	Age at Tx > 45 years	1.04	(1.00–1.07)	0.044 *	Age at Tx > 45 years	1.02	(0.98–1.05)	0.357
Retransplantation	0.96	(0.29–3.2)	0.95	Retransplantation	2.46	(0.29–21.12)	0.412	Retransplantation	0.46	(0.1–2.04)	0.306
Monotherapy	2.18	(0.81–5.84)	0.12	Monotherapy	NA	NA	NA	Monotherapy	1.11	(0.41–3.06)	0.835
Double drug therapy	0.88	(0.46–1.67)	0.69	Double drug therapy	0.56	(0.19–1.61)	0.282	Double drug therapy	1.18	(0.51–2.75)	0.695
Triple drug therapy	0.97	(0.51–1.86)	0.934	Triple drug therapy	1.79	(0.62–5.14)	0.282	Triple drug therapy	0.91	(0.35–2.36)	0.851
GCS	0.87	(0.43–1.78)	0.703	GCS	0.68	(0.18–2.59)	0.574	GCS	1.66	(0.7–3.94)	0.247
CsA	0.44	(0.20–0.95)	0.036 *	CsA	0.59	(0.21–1.64)	0.31	CsA	4.55	(0.91–22.8)	0.065
TAC	5.09	(2.00–12.95)	<0.001 *	TAC	6.25	(1.68–23.21)	0.006 *	TAC	0.22	(0.04–1.1)	0.065
AZA	0.68	(0.28–1.64)	0.385	AZA	1.28	(0.42–3.95)	0.664	AZA	0.58	(0.08–4.39)	0.596
MMF	0.78	(0.41–1.5)	0.463	MMF	1.04	(0.36–3.05)	0.936	MMF	0.68	(0.28–1.64)	0.399
ATG induction	0.33	(0.04–2.52)	0.288	ATG induction	0.48	(0.06–3.8)	0.488	ATG induction	NA	NA	NA
Anti-CD25 induction	1.35	(0.69–2.64)	0.376	Anti-CD25 induction	0.74	(0.2–2.67)	0.645	Anti-CD25 induction	1.07	(0.44–2.6)	0.884
AR treated with GCS	0.75	(0.37–1.51)	0.413	AR treated with GCS	0.61	(0.18–2.01)	0.413	AR treated with GCS	0.72	(0.3–1.76)	0.476
AR treated with ATG	1.38	(0.18–10.37)	0.753	AR treated with ATG	NA	NA	NA	AR treated with ATG	0.9	(0.12–6.93)	0.922
EBV DNA	0.72	(0.31–1.68)	0.447	EBV DNA	0.7	(0.08–6.03)	0.744	EBV DNA	1.66	(0.62–4.43)	0.314
CMV DNA	0.6	(0.14–2.51)	0.482	CMV DNA	1.26	(0.27–5.87)	0.767	CMV DNA	NA	NA	NA
HBV DNA	0.86	(0.26–2.83)	0.802	HBV DNA	0.61	(0.08–4.78)	0.638	HBV DNA	1.32	(0.3–5.85)	0.718
HCV RNA	1.08	(0.45–2.61)	0.861	HCV RNA	0.48	(0.06–3.8)	0.488	HCV RNA	0.96	(0.35–2.65)	0.942
SOT—Multivariate				KTR—Multivariate				LTR—Multivariate			
Variable	HR	95% CI	*p*-Value	Variable	HR	95% CI	*p*-Value	Variable	HR	95% CI	*p*-Value
LTx	1.75	(0.63–4.83)	0.281	Male sex	7.87	(1.25–49.26)	0.027 *	Male sex	0.872	(0.36–2.13)	0.746
Age at Tx > 45 years	3.16	(1.4–7.1)	0.005 *	Age at Tx > 45 years	5.21	(1.04–26.25)	0.045 *	Age at Tx > 45 years	1.61	(0.62–4.21)	0.332
TAC	5.85	(0.89–38.24)	0.065	TAC	18.57	(2.79–123.87)	0.003 *	TAC	0.24	(0.04–1.35)	0.107
CsA	1.91	(0.39–9.31)	0.423								

AR—acute rejection, ATG—anti-thymocyte globulin, AZA—azathioprine, CMV—cytomegalovirus, CsA—cyclosporin, EBV—Epstein-Barr virus, GCs—glucocorticosteroids, HBV—hepatitis B virus, HCV—Hepatitis C virus, KT+LTRs—non-simultaneous kidney and liver transplant recipients, KTRs—kidney transplant recipients, LTRs—liver transplant recipients, LTx—liver transplantation, MMF—mycophenolate mofetil, SOT—solid organ transplantation group, TAC—tacrolimus, Tx—transplantation. Wald test’s *p*-values. * *p* < 0.05, NA—not applicable.

**Table 4:** PTLD treatment.

	LTRs	KTRs	
PTLD Treatment	*n* (%)		*p*-Value
RIS RAPA	22 (95.7)	14 (87.5)	0.37
	9 (39.1)	3 (18.8)	0.186
EVR Anti-CD20	0 (0)	1 (6.3)	0.251
	6 (26.1)	4 (25)	0.955
Chemotherapy -R-CHOP	14 (60.9)	11 (68.8)	0.631
	7 (30.4)	2 (12.5)	0.204
-CHOP	4 (17.4)	2 (12.5)	0.698
-Other	3 (13)	7 (43.8)	0.02 *
Surgery	8 (34.8)	5 (31.3)	0.834
Radiotherapy	0 (0)	2 (12.5)	0.093

Chemotherapy—combined instances R-CHOP, CHOP, and other use, EVR—conversion to everolimus, KTRs—kidney transplant recipients, LTRs—liver transplant recipients, Other—chemotherapy protocols less frequently used in PTLD (e.g., doxorubicin, bleomycin, vinblastine, dacarbazine (ABVD), ifosfamide, carboplatin, etoposide (ICE), cyclophosphamide monotherapy), RAPA—conversion to sirolimus, RIS—reduction of immunosuppression, R-CHOP—anti-CD20 + CHOP. *p*-values represent the Mann–Whitney U-test comparison between KTRs and LTRs. * *p* < 0.05.

**Table 5:** Outcomes.

	LTRs	KTRs	
Outcomes	*n* (%)		*p*-Value
CR	12 (52.2)	3 (18.8)	0.039 *
Mean CR duration (months) (SD) Mean survival (months) (SD)	73.7 (56.1)	89.7 (61.7)	0.711
	56.1 (53.4)	37.3 (51.9)	0.28
Total deaths PTLD related deaths	9 (39.1)	6 (37.5)	0.933
	5 (21.7)	4 (25)	0.83
Alive	12 (52.2)	9 (56.3)	0.817
Lost to follow up	2 (8.7)	1 (6.3)	0.805

CR—complete remission. KTRs—kidney transplant recipients, LTRs—liver transplant recipients. *p*-values represent the Mann-Whitney U-test comparison between KTRs and LTRs. * *p* < 0.05.

## Data Availability

The data presented in this study are available upon request by the authors.

## Supplementary Materials

The following supporting information can be downloaded at: https://www.mdpi.com/article/10.3390/cancers14081953/s1, Table S1: History of immunosuppressive treatment in our cohort of PTLD patients; Table S2: PTLD location and dissemination in the SOT group (*n* = 39).
