# Supplementary material for: Comparison of Post-Transplantation Lymphoproliferative Disorder Risk and Prognostic Factors between Kidney and Liver Transplant Recipients
Source: Cancers (Basel). 2022 Apr 13;14(8):1953. doi: 10.3390/cancers14081953 (PMC9024969; doi:10.3390/cancers14081953)
Supplement: Supplementary file 1 [file cancers-14-01953-s001.zip › Supplement Table S1.pdf]

Patient number	SOT type	IS regimen at PTLD diagnosis	Mean CsA concentration (ng/mL)	CsA duration (months)	Mean TAC concentration (ng/mL)	TAC duration (months)	Total MMF consumed (g)	Total AZA consumed (g)	Total GCS administered/ consumed (g of prednisone)	Number of IS conversions prior to PTLD	Time between last IS conversion and PTLD (months)
1	KTX	GCS+TAC+AZA	0	0	11.71	22.27	0	68.3	13.13	0	NA
2	KTX	GCS+TAC+MMF	0	0	6.77	117.9	853	0	2.28	0	NA
3	KTX	GCS+AZA	183.26	117.03	0	0	3646.5	955.5	61.65	5	4.23
4	KTX	TAC+AZA	0	0	14.24	64.6	0	196.8	1.83	1	60.37
5	KTX	GCS+CsA+MMF	168.81	222.87	0	0	4867.76	402.7	64.78	1	102.27
6	KTX	CsA+MMF	92.24	174.57	0	0	8961	0	9.8	1	121.9
7	KTX	GCS+MMF	165.18	19.9	0	0	12352.5	25.05	56.99	2	191.67
8	KTX	CsA+MMF	139.97	156	0	0	7798	81.7	31.41	2	63.77
9	KTX	GCS+TAC+MMF	0	0	14.35	5.4	237.6	0	2.54	0	NA
10	KTX	GCS+CsA+MMF	189.14	40.4	0	0	339	81.45	18.28	1	6.73
11	KTX	GCS+CsA+MMF	138.21	123.93	NA	2.5	7710	0	20.36	1	123.93
12	KTX	GCS+TAC+MMF	0	0	10.5	37.67	1147	0	5.25	0	NA
13	KTX	GCS+CsA									
14	KTX	GCS+CsA+AZA									
15	KTX	GCS+TAC+AZA									
16	KTX	GCS+CsA+MMF									
17	LTX	GCS+TAC+MMF	0	0	10.81	10.93	141	0	2.21	1	10.37
18	LTX	GCS+TAC	0	0	19.08	3.47	0	0	1.03	0	NA
19	LTX	GCS+CsA	119.15	2.43	12.2	24.6	13	0	9.38	2	21.77
20	LTX	GCS+TAC+MMF	0	0	8.8	25.5	534	0	6.27	0	NA
21	LTX	GCS+TAC	0	0	10.37	36.7	0	0	11.16	0	NA
22	LTX	GCS+TAC	0	0	12.06	30.47	128	0	14.35	1	26.27
23	LTX	TAC	0	0	9.33	60.5	0	0	7.11	1	33.6
24	LTX	GCS+TAC+MMF	0	0	9.86	63.1	1289.75	0	17.65	0	NA
25	LTX	GCS+TAC	213.57	6.87	11.68	82.73	1231	0	28.32	2	44.07
26	LTX	TAC+MMF	0	0	11.47	70.63	98	0	4.49	3	3.7
27	LTX	GCS+TAC+MMF	0	0	6.91	161.97	4927	0	24.56	0	NA
28	LTX	TAC+MMF	0	0	9.72	45.03	837	0	0	0	NA
29	LTX	TAC+MMF	0	0	6.58	134.53	3332.75	0	16.35	3	21.2
30	LTX	GCS+TAC+AZA	0	0	10.83	71.83	0	51.15	27.91	1	11.23
31	LTX	GCS+CsA	157.08	4.23	9.9	1.34	0	0	2.19	1	4.23
32	LTX	TAC	0	0	11.7	37.23	0	0	3.5	1	24.07
33	LTX	TAC	0	0	13.57	32.1	0	0	9.38	1	10
34	LTX	GCS+TAC+MMF	0	0	9.49	7.53	102	0	3.12	1	6.97
35	LTX	GCS+TAC	0	0	12.79	16.63	0	0	4.7	0	NA
36	LTX	TAC+MMF	0	0	8.19	53.7	1765	0	0.77	1	43.73
37	LTX	TAC+MMF	0	0	6.7	68.53	662	0	4.61	2	16.27
38	LTX	TAC									
39	LTX	GCS+TAC+MMF									

Supplement Table S1. History of immunosuppressive treatment in our cohort of PTLD patients. AZA- azathioprine, CsA – cyclosporin, GCS –glucocorticosteroids, KTX – kidney transplant, LTX – liver transplant, MMF – mycophenolate mofetil, SOT – solid organ transplant, TAC – tacrolimus, NA – not applicable. In four KTRs and two LTRs detailed data about IS conversions, dose modifications was unavailable due to missing or incomplete medical documentation (marked gray).
