# Supplementary material for: Comparison of Post-Transplantation Lymphoproliferative Disorder Risk and Prognostic Factors between Kidney and Liver Transplant Recipients
Source: Cancers (Basel). 2022 Apr 13;14(8):1953. doi: 10.3390/cancers14081953 (PMC9024969; doi:10.3390/cancers14081953)
Supplement: Supplementary file 1 [file cancers-14-01953-s001.zip › Supplement Table S2.pdf]

Location	Number of patients
Lymph node	19
Bone marrow	12
Colon	5
Liver	6
Spleen	7
Tonsil	6
Lung	4
Small intestine	4
Kidney	3
Pancreas	1
Brain	1
Cerebrospinal flui	1
Skin	1
Calcaneus	1
**Dissemination**	**Number of patients**
Focal (1 location)	21
2 locations	10
3 locations	5
4 locations	0
5 locations	2
6 locations	1

Supplement Table S2. PTLD location and dissemination in the SOT group (n=39).
